# A Highly Polymorphic Receptor Governs Many Distinct Self-Recognition Types within the *Myxococcales* Order

**DOI:** 10.1128/mBio.02751-18

**Published:** 2019-02-12

**Authors:** Pengbo Cao, Xueming Wei, Ram Prasad Awal, Rolf Müller, Daniel Wall

**Affiliations:** a Department of Molecular Biology, University of Wyoming, Laramie, Wyoming, USA; b Department of Microbial Natural Products, Helmholtz Institute for Pharmaceutical Research Saarland, Helmholtz Centre for Infection Research and Department of Pharmacy, Saarland University, Saarbrücken, Germany; Princeton University

**Keywords:** cell surface, cellular transfer, kin recognition, myxobacteria, polymorphism

## Abstract

Self-recognition underlies sociality in many group-living organisms. In bacteria, cells use various strategies to recognize kin to form social groups and, in some cases, to transition into multicellular life. One strategy relies on a single genetic locus that encodes a variable phenotypic tag (“greenbeard”) for recognizing other tag bearers. Previously, we discovered a polymorphic cell surface receptor called TraA that directs self-identification through homotypic interactions in the social bacterium Myxococcus xanthus. Recognition by TraA leads to cellular resource sharing in a process called outer membrane exchange (OME). A second gene in the *traA* operon, *traB*, is also required for OME but is not involved in recognition. Our prior studies of TraA identified only six recognition groups among closely related M. xanthus isolates. Here we hypothesize that the number of *traA* polymorphisms and, consequently, the diversity of recognition in wild isolates are much greater. To test this hypothesis, we expand the scope of TraA characterization to the order *Myxococcales*. From genomic sequences within the three suborders of *Myxococcales*, we identified 90 *traA* orthologs. Sequence analyses and functional characterization of *traAB* loci suggest that OME is well maintained among diverse myxobacterial taxonomic groups. Importantly, TraA orthologs are highly polymorphic within their variable domain, the region that confers selectivity in self-recognition. We experimentally defined 10 distinct recognition groups and, based on phylogenetic and experimental analyses, predicted >60 recognition groups among the 90 *traA* alleles. Taken together, our findings revealed a widespread greenbeard locus that mediates the diversity of self-recognition across the order *Myxococcales*.

## Abstract (precis)

Many biological species distinguish self from nonself by using different mechanisms. Higher animals recognize close kin via complex processes that often involve the five senses, cognition, and learning, whereas some microbes achieve self-recognition simply through the activity of a single genetic locus. Here we describe a single locus, *traA*, in myxobacteria that governs cell-cell recognition within natural populations. We found that *traA* is widespread across the order *Myxococcales*. TraA is highly polymorphic among diverse myxobacterial isolates, and such polymorphisms determine selectivity in self-recognition. Through bioinformatic and experimental analyses, we showed that *traA* governs many distinct recognition groups within *Myxococcales*. This report provides an example in which a single locus influences social recognition across a wide phylogenetic range of natural populations.

## INTRODUCTION

Bacteria are excellent team players and have evolved numerous ways to exploit group cooperation to survive in rapidly changing environments. Self-recognition is an important survival strategy that allows individuals to establish social contacts with close kin and conduct multicellular behaviors (1). Recognition in bacteria often relies on the matching of specific loci that indicate genetic relatedness between cells (2). Selectivity in self-recognition is enabled by genetic polymorphisms within these loci. In nature, bacteria are vastly diverse. However, little is known about the conservation and variability of bacterial recognition systems among wild populations. In addition, how polymorphic loci govern self-recognition across a wide phylogenetic range of natural isolates remains underexplored.

Bacteria use specific cues (e.g., diffusible chemicals, cell surface receptors) to communicate with their neighbors and recognize kin (1, 3). One example of self-recognition that we recently described is in a Gram-negative soil-dwelling bacterium, Myxococcus xanthus. Myxobacteria are well known for their sociality, which is exemplified by their ability to aggregate individuals from the environment and build multicellular fruiting bodies wherein cells differentiate into spores in response to starvation (4). To help modulate their social life, myxobacteria are equipped with recognition and discrimination systems. M. xanthus uses a polymorphic cell surface receptor, TraA, to recognize related individuals upon physical contact (5). This system is termed outer membrane exchange (OME), because bulk sharing of outer membrane (OM) components between cells occurs after recognition. The sharing of OM materials among related individuals facilitates cooperation, including the repair of damaged membranes or the phenotypic restoration of cellular defects caused by mutations (6). In contrast, if two individuals share compatible TraA receptors but are not true clonemates, then antagonistic interactions can ensue (7, 8).

Two OM proteins, TraA and TraB (TraA/B), play indispensable roles in OME (9). TraA/B function together as cell-cell adhesins. TraA is a cell surface receptor and, notably, is polymorphic within its variable domain (VD) across environmental isolates (5). These polymorphisms dictate selective cell-cell interactions, and only individuals bearing identical or nearly identical TraA receptors recognize others as self and undergo OME. Although TraB does not determine the specificity of self-recognition, it assists TraA to function in cell-cell binding and OME (10). We previously showed that coincubation of M. xanthus strains expressing different TraA receptors leads to selective cell-cell adhesion in mixed populations (10), indicating that TraA homotypic interactions govern self-recognition between cells. We also revealed the malleability of TraA recognition (10). Sequence changes within the VD can alter the TraA recognition specificity and subsequently reprogram how cells interact.

A simple and elegant recognition system that is derived from kin selection theory relies upon a single genetic locus and is called “greenbeard” recognition (11–13). The M. xanthus *traA* locus fulfills the criteria for identification as a greenbeard gene (14), as it (i) encodes a perceptible cue, (ii) allows recognition of others bearing the same cue, and (iii) facilitates preferential interactions among the cue-bearers. Polymorphic greenbeard loci are thought to be rare in nature, and one reason is that their genetic diversity is not easily maintained during evolution (15). In theory, the most common allele(s) is predicted to reach fixation because it provides a fitness advantage to the largest population, whereas rare alleles are eliminated because they provide fitness gains only infrequently. However, because OME among nonclonemates involves antagonistic outcomes, there is selective pressure for the maintenance and diversification of TraA recognition in OME, mediated by polymorphisms within the VD (7).

Our previous studies on 16 isolates of M. xanthus revealed six distinct recognition groups, named A through F (5, 10). Another study, using some of the same strains and additional strains all isolated from the same small patch of soil, inferred that those *traA* alleles belonged within the same recognition groups by bioinformatic analysis (16). Because those prior studies of TraA diversity were limited to a single species and were biased to include highly related strains, we hypothesized that the scope of TraA variability, and hence recognition diversity, was largely unexplored. Therefore, to expand our understanding of the TraA diversity landscape, we examined 90 wild-type alleles across the *Myxococcales* order. We evaluated the prevalence of OME in *Myxococcales* through the identification and, in some cases, the characterization of *traAB* loci from these isolates. Using phylogenetic and experimental approaches, we show that TraA is a highly polymorphic locus that governs self-recognition among the members of a diverse collection of natural myxobacterial isolates.

## RESULTS

### Identification of *traAB* alleles across the *Myxococcales* order.

**FIG 1:**
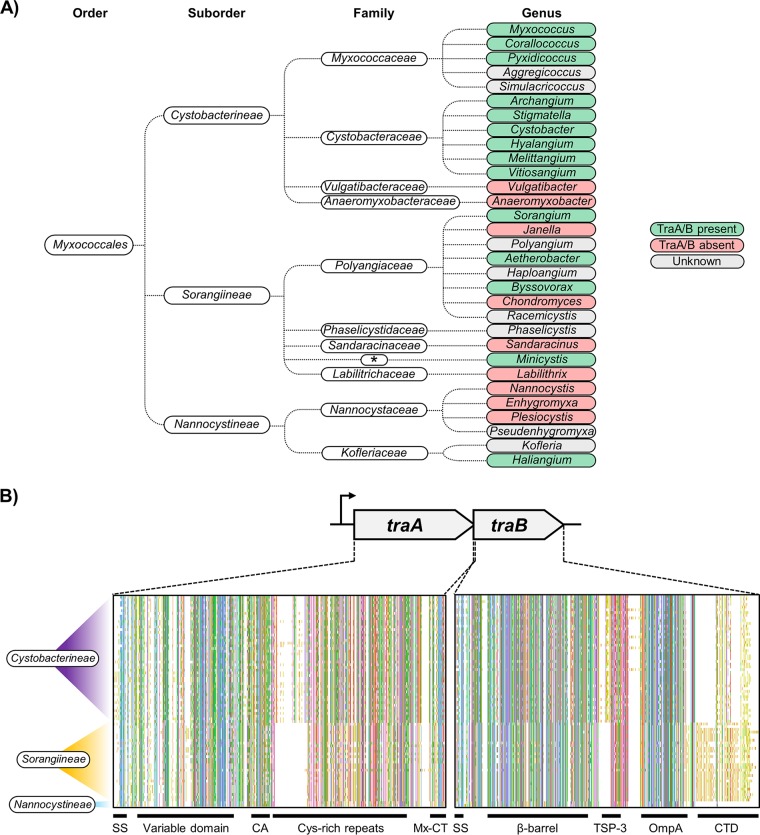
Identification of *traAB* orthologs across the order *Myxococcales*. (A) The monophyletic order *Myxococcales* currently contains three well-defined suborders, 10 families, and 31 genera. Genera that harbor or lack *traAB* orthologs are indicated, whereas genera with unavailable genomic sequences are shaded in gray. *, family affiliation not designated. (B) Schematics of multiple-sequence alignments of 78 TraA/B orthologs across the order *Myxococcales*. Amino acid residues are displayed using the Clustal X default scheme. The *traAB* operon is shown at the top. Domain architectures of TraA/B are labeled as follows: SS, type I signal sequence; CA, Cys-A region; Mx-CT, MYXO-CTERM (TIGR03901); TSP-3, thrombospondin type 3 (Pfam02412); CTD, carboxyl-terminal domain. Suborders of the aligned TraA/B sequences are indicated on the left.

To date, orthologs of *traAB* have been found only within the order *Myxococcales*, a highly diverse group of social bacteria within the *Deltaproteobacteria* class. To assess the prevalence of OME in this order, we identified *traAB* orthologs from a wide variety of natural *Myxococcales* isolates from in-house and public databases. Currently, the *Myxococcales* order contains 31 known genera (62 species) (17–19), 23 of which have draft or complete genomic sequences that were usable in this study. Inspection of these genomes from over 100 isolates, combined with 12 *traA* alleles that we had previously sequenced (5), identified 90 *traA* genes (89 of which were unique). For all of the *traA* alleles found from genomic searches, we also identified the corresponding *traB* locus; 76 of the alleles were unique. These isolates encompassed all 3 suborders and included 4 families, 14 genera, 25 defined species, and 9 operational taxonomic units (currently with no species description) (Fig. 1A; see also Fig. S1 in the supplemental material).

**FIG 2:**
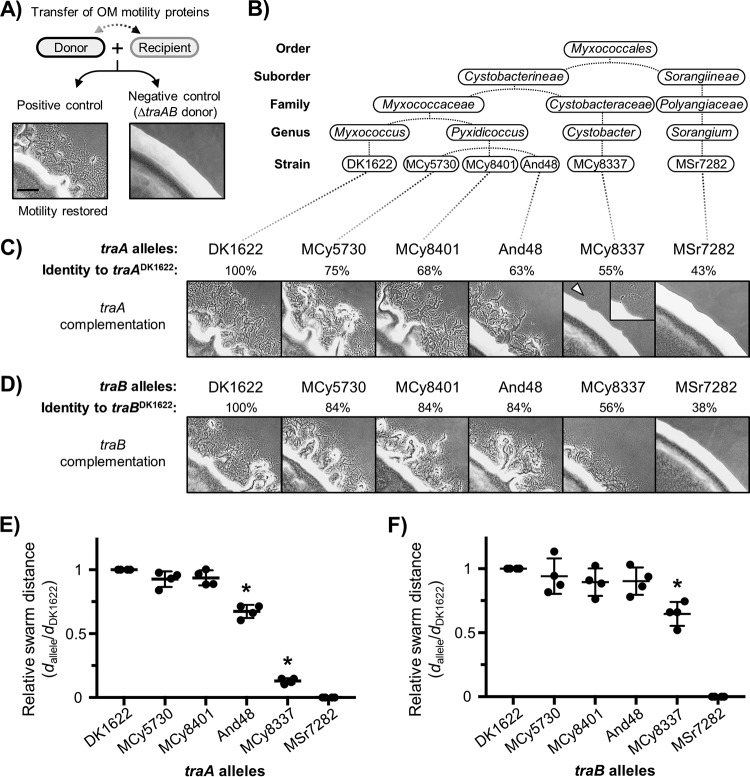
Functional characterization of diverse *traAB* orthologs in *Myxococcales*. (A) Schematic of the stimulation assay that restores motility to certain mutants (recipients) by the transfer of wild-type motility proteins from nonmotile donors. A positive control (donor and recipient, with both harboring *traAB*^DK1622^) and a negative control (donor lacking *traAB*) are shown. (B) Taxonomic origins of the *traAB* alleles analyzed. (C) Stimulation assays testing for functional complementation by heterologously expressing different *traA* alleles in isogenic Δ*traA* M. xanthus strains. Each micrograph shows a mixture of donors and recipients bearing identical *traA* alleles. Allele names and their percentages of identical amino acids relative to *traA*^DK1622^ (full length) are shown. The arrowhead highlights a small emergent flare at the edge of the colony. The inset shows an enlarged view of stimulated cells. (D) Stimulation assays testing for complementation of heterologous *traB* alleles in isogenic Δ*traB* strains. Allele names and allele identities to *traB*^DK1622^ are indicated. Stimulation efficacy was calculated by measuring the distance (*d*) of the movement of emergent flares from colony edges. Data representing the relative swarming distances determined in *traA* and *traB* complementation experiments are shown in panels E and F, respectively. Four experimental replicates were done. Error bars represent standard deviations from the means. Significant differences between the DK1622 group and other groups (i.e., functionally distant alleles) are indicated by asterisks (*P < *0.05 [*t* test]). Strain details are given in Table 2 (see also Table S1). Scale bar, 200 µm.

10.1128/mBio.02751-18.1 FIG S1 Phylogenies of TraA and TraB orthologs identified across the order *Myxococcales*. Maximum likelihood trees are shown for 90 TraA orthologs (top) and 79 TraB orthologs (bottom). Different suborders, families, and genera are labeled with distinct border lines and colors. Allele prefixes indicate taxonomic origin (refer to Table 1). The A/P205 molecular switch residues are also labeled. Red stars highlight the alleles tested in Fig. 2. Branch supports are color coded. Scale bars represent the number of substitutions per residue. Download FIG S1, TIF file, 1.8 MB. Copyright © 2019 Cao et al. 2019 Cao et al. This content is distributed under the terms of the Creative Commons Attribution 4.0 International license.

Some myxobacteria lacked *traAB* orthologs. For example, *Anaeromyxobacter* and *Vulgatibacter* isolates lacked *traAB*, but as species from these genera have significantly reduced genome sizes—about half the size of the genomes of typical myxobacteria—and have lost many of their social traits (20–23), their absence from these genera was not surprising. In other cases, myxobacteria that have large genomes and complex social behaviors (e.g., Chondromyces crocatus) also lacked *traAB* orthologs (24). As we considered it plausible that analogs of TraA/B could function in OME, we experimentally tested whether the C. crocatus Cm c5 strain could undergo OME with clonemates. However, using a lipophilic dye transfer assay (9), we did not detect transfer (Fig. S2), which, in conjunction with the absence of TraA/B, suggests that this strain does not undergo OME.

10.1128/mBio.02751-18.2 FIG S2 Fluorescent marker transfer assay performed with M. xanthus, S. cellulosum, and C. crocatus cells. (A) Cells labeled with Cy3 (donors) were mixed with the same strain labeled with CFDA-SE (recipients). M. xanthus strains DK8615 and DW1415 served as experimental positive and negative controls, respectively. Cells for which transfer of Cy3-labeled material occurred are indicated with arrowheads. (B) A lipid dye transfer assay (9) was used to test for OME in C. crocatus cells. Donor cells were labeled with red fluorescent DiD lipid dye, and recipient cells were labeled with CFDA-SE. Scale bar, 3 µm. Download FIG S2, TIF file, 4.2 MB. Copyright © 2019 Cao et al. 2019 Cao et al. This content is distributed under the terms of the Creative Commons Attribution 4.0 International license.

The discovery of *traAB* across a wide spectrum of *Myxococcales* natural isolates suggests that OME was relatively well maintained during evolution (Fig. 1A). To explore the evolutionary relationships of these orthologs, we constructed phylogenetic trees of TraA and TraB (Fig. S1). Prefixes were assigned to *traAB* allele in the trees to indicate their taxonomic origins (see Table 1). TraA/B orthologs from within and related species were found to cluster together, and the TraA/B phylogenies were generally congruent with myxobacteria taxonomy (refer to Fig. 1A), suggesting that the *traAB* genes were primarily inherited by vertical transmission.

**TABLE 1:** Abbreviations used as prefixes to *traAB* alleles that indicate taxonomic origins

Prefix	Species
Aef	*Aetherobacter fasciculatus*
Aer	*Aetherobacter rufus*
AeC^*a*^	*Aetherobacter* clade
Ar	*Archangium gephyra*
ArC^*a*^	*Archangium* clade
Byc	*Byssovorax cruenta*
Ccc	*Corallococcus coralloides*
CcC^*a*^	*Corallococcus* clade
Cba	*Cystobacter armeniaca*
Cbfe	*Cystobacter ferrugineus*
Cbfu	*Cystobacter fuscus*
Cbv	*Cystobacter velatus*
Cbvi	*Cystobacter violaceus*
CbC^*a*^	*Cystobacter* clade
Hao	*Haliangium ochraceum*
Hym	*Hyalangium minutum*
HyC^*a*^	*Hyalangium* clade
Meb	*Melittangium boletus*
Mir	*Minicystis rosea*
Mxf	*Myxococcus fulvus*
Mxh	*Myxococcus hansupus*
Mxm	*Myxococcus macrosporus*
Mxst	*Myxococcus stipitatus*
Mxv	*Myxococcus virescens*
Mxx	*Myxococcus xanthus*
MxC^*a*^	*Myxococcus* clade
MyxC^*a*^	*Myxococcaceae* clade
Pxf	*Pyxidicoccus fallax*
PxC^*a*^	*Pyxidicoccus* clade
SoS^*a*^	*Sorangiineae* suborder
Soce	*Sorangium cellulosum*
Sga	*Stigmatella aurantiaca*
Sge	*Stigmatella erecta*

a Unclassified species that is assigned to a defined clade (C) or suborder (S) based on 16S rRNA sequence similarities.

The prototypic TraA proteins from M. xanthus isolates harbor an N-terminal VD (a distant homolog of the PA14 domain [Pfam07691]) followed by a more conserved C-terminal region that contains cysteine-rich repeats and a putative protein sorting motif called MYXO-CTERM (9). The prototypic TraB proteins harbor an N-terminal OM β-barrel domain and a C-terminal region that contains thrombospondin type 3 repeats and an OmpA domain (10). A multiple-sequence alignment of distant TraA/B orthologs shows that they have the same domain architectures as the M. xanthus TraA/B proteins (Fig. 1B). However, the patterns of sequence conservation of TraA/B orthologs did show some variation across the *Myxococcales* order, which prompted us to evaluate whether they share functions similar to those of the M. xanthus TraA/B proteins.

### Functional analysis of *traAB* alleles from distant myxobacterial isolates.

To examine whether TraA/B orthologs are functional in OME, we selected five distinct allele sets for heterologous expression in isogenic M. xanthus strains that lack their native *traA* or *traB* genes. An extracellular complementation assay named “stimulation” was used to assess whether these divergent alleles can restore OME to these M. xanthus mutants (9). In brief, this assay monitors the outcome of OME between two mutants with different motility defects (25, 26). Here, the transfer of OM lipoproteins from a nonmotile donor strain to a nonmotile recipient strain led to the restoration of motility to the recipients by providing the missing motility proteins, which was observed as emergent flares from the edge of the mixed colony (Fig. 2A). First, we expressed divergent *traA* alleles in the Δ*traA* donor and a cognate Δ*traA* recipient strain (note that these *tra* mutants were derived from the M. xanthus DK1622 laboratory strain) and tested for functional complementation (see Fig. 2B and Table 2 for details). As shown in Fig. 2C, TraA^MCy5730^, TraA^MCy8401^, TraA^And48^, and TraA^MCy8337^ were all functional in this assay whereas TraA^MSr7282^ was nonfunctional.

**TABLE 2:** Taxonomic origins of the *traAB* alleles tested by stimulation assay

Allele	Taxonomic description		Strains^*a*^	Experimental use
	Genus	Species		
DK1622	*Myxococcus*	*xanthus*	*traA*^DK1622^ D (DK8601), *traA*^DK1622^ R (DW1466)	Fig. 2A, C, and D, 5C, and 6A
DK816	*Myxococcus*	*xanthus*	*traA*^DK816^ D (DW1468), *traA*^DK816^ R (DW2221)	Fig. 6A
Pali	*Myxococcus*	*xanthus*	*traA*^Pali^ D (DW1471), *traA*^Pali^ R (DW2224)	Fig. 6A
A96	*Myxococcus*	*xanthus*	*traA*^A96^ D (DW1469), *traA*^A96^ R (DW2222)	Fig. 6A
DK805	*Myxococcus*	*xanthus*	*traA*^DK805^ D (DW2212), *traA*^DK805^ R (DW2234)	Fig. 6A
Mf(HW-1)	*Myxococcus*	*fulvus*	*traA*^Mf^ D (DW1470), *traA*^Mf^ R (DW2223)	Fig. 6A
MCy5730	*Pyxidicoccus*	Unclassified	*traA*^MCy5730^ D (DW2243), *traA*^MCy5730^ R (DW2248), *traB*^MCy5730^ D (DW2257)	Fig. 2C and D, 5C, and 6A
MCy8401	*Pyxidicoccus*	Unclassified	*traA*^MCy8401^ D (DW2244), *traA*^MCy8401^ R (DW2249), *traB*^MCy8401^ D (DW2258)	Fig. 2C and D, 5C, and 6A
And48	*Pyxidicoccus*	*fallax*	*traA*^And48^ D (DW2245), *traA*^And48^ R (DW2254), *traB*^And48^ D (DW2259)	Fig. 2C and D and 6A
MCy8337	*Cystobacter*	*violaceus*	*traA*^MCy8337^ D (DW2246), *traA*^MCy8337^ R (DW2255), *traB*^MCy8337^ D (DW2260)	Fig. 2C and D
MSr7282	*Sorangium*	*cellulosum*	*traA*^MSr7282^ D (DW2247), *traA*^MSr7282^ R (DW2256), *traB*^MSr7282^ D (DW2261)	Fig. 2C and D

a Strains tested in stimulation assays were constructed by expressing different *traA* or *traB* alleles in isogenic Δ*traA* or Δ*traB* strains derived from M. xanthus DK1622. D, donor; R, recipient. See Table S1 for additional details.

10.1128/mBio.02751-18.8 TABLE S1 Plasmids and strains used in this study. Download Table S1, DOCX file, 0.03 MB. Copyright © 2019 Cao et al. 2019 Cao et al. This content is distributed under the terms of the Creative Commons Attribution 4.0 International license.

We next evaluated the corresponding *traB* alleles by expressing them in a M. xanthus Δ*traB* donor strain that harbors its native *traA*^DK1622^ allele. After mixing of the donors (refer to Table 2 for strain details) with the *traAB*^DK1622^ recipient, TraB^MCy5730^, TraB^MCy8401^, TraB^And48^, and TraB^MCy8337^ were all found to complement the OME defect of the Δ*traB* strain (Fig. 2D). As previously reported (10), this result confirms that TraB does not contribute to the specificity of recognition, because the donor and recipient strains contained different *traB* alleles but the same *traA* allele. In contrast to the other four *traB* alleles, TraB^MSr7282^ was nonfunctional, as was similarly found for TraA^MSr7282^. Because *traAB* from Sorangium cellulosum MSr7282 was more divergent from the other four myxobacteria isolates (Fig. S1), we postulated that the S. cellulosum TraA/B proteins may not interact with the cognate M. xanthus DK1622 counterparts. To address this possibility, we cloned the entire *traAB* operon from S. cellulosum MSr7282 and placed it in Δ*traAB* M. xanthus donor and recipient strains. However, OME was again not restored (data not shown).

The abilities of *traAB* orthologs to functionally complement OME differed, as judged from the degree of stimulation (Fig. 2E and F). Such variation correlated with the phylogenetic distance between these orthologs and *traAB* from DK1622 (Fig. S1). That is, the *traAB*^MCy8401^ and *traAB*^MCy5730^ allele sets were the most similar to *traAB*^DK1622^, and they restored OME in M. xanthus to a level comparable to that seen with the native alleles. A more distant allele, *traA*^MCy8337^, showed poor functional complementation in M. xanthus (Fig. 2C, inset). To clearly assess specificity in recognition (see descriptions of experiments below), a chimeric allele was created where VD^MCy8337^ was fused with the C terminus of TraA^DK1622^ (Fig. S3), with the goal of improving activity. As predicted, the resulting allele showed robust functional complementation in testing against itself in M. xanthus, indicating that the C terminus of TraA, although not involved in recognition specificity, is important for OME and for interaction with host components. Consistent with the *traA*^MCy8337^ finding, the complementation of OME by *traB*^MCy8337^ was also relatively poor compared with that seen with the other functional alleles (Fig. 2F).

10.1128/mBio.02751-18.3 FIG S3 Construction of a chimeric allele that harbors VD^MCy8337^. (A) Schematics showing the parental TraA^DK1622^ and TraA^MCy8337^ alleles and the chimeric allele for which the VD from TraA^MCy8337^ was swapped into TraA^DK1622^. Dashed underlines indicate the TraA fragments used to create the chimeric allele. Protein lengths are shown on the right. (B) Stimulation assays testing both parental alleles and the chimeric allele in which each allele was placed in isogenic donor/recipient strain sets. White arrowheads indicate small emergent flares. The inset shows an enlarged view of emergent flares. The chimeric *traA*^MCy8337/DK1622^ allele displayed enhanced stimulation compared with *traA*^MCy8337^ (a second copy of *traB*^DK1622^ was also introduced into the donor/recipient strains to increase stimulation levels without altering recognition specificity). Strain details are given in Table S1. Scale bar, 200 µm. Download FIG S3, TIF file, 0.9 MB. Copyright © 2019 Cao et al. 2019 Cao et al. This content is distributed under the terms of the Creative Commons Attribution 4.0 International license.

S. cellulosum *traAB*^MSr7282^ represents one of the alleles that are most distant from *traAB*^DK1622^, and it failed to complement OME in M. xanthus. Importantly, M. xanthus and S. cellulosum are in different suborders (Fig. 1A), and they have major physiological differences (27). For instance, S. cellulosum has an atypical OM that lacks lipopolysaccharides (LPS) (28), whereas M. xanthus and other species within the *Cystobacterineae* suborder have OMs that contain LPS. Because TraA resides on the cell surface and TraB resides in the OM, these differences in OM lipid composition complicate their functional analysis. Therefore, to test for OME, we developed an alternative strategy to detect transfer in S. cellulosum by directly labeling the surface proteins with the fluorescent Cy3 dye. As an experimental control, M. xanthus cells showed robust transfer of Cy3-labeled surface proteins to recipient cells in a *tra*-dependent manner (Fig. S2). We tested two S. cellulosum isolates under similar conditions, but no transfer was conclusively detected (Fig. S2). These findings suggest that S. cellulosum conducts OME only under particular/regulated conditions or that the *traAB* genes have an alternative function in this species.

**FIG 3:**
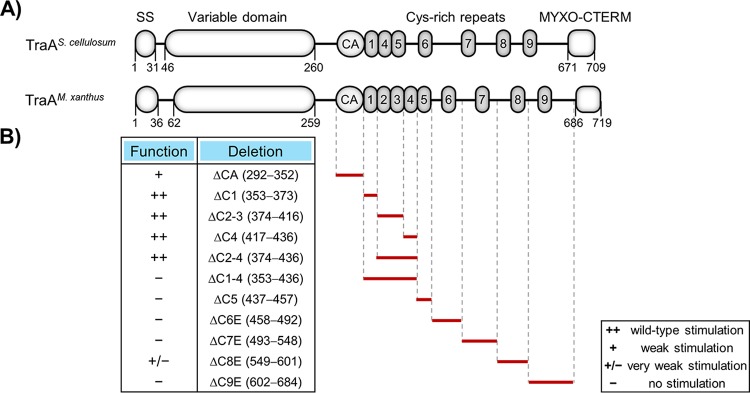
Deletion analysis of the Cys-rich region within TraA^DK1622^. (A) Domain architectures of TraA from a S. cellulosum strain (MSr7282) and a M. xanthus strain (DK1622). Cys-rich repeats are numerically labeled; note that S. cellulosum is missing C2 and C3. (B) Schematic depicting markerless in-frame deletion mutants (deleted residues listed) within the Cys-rich region of TraA^DK1622^. The ability of these mutants to complement a Δ*traA* mutant was assessed by a stimulation assay. Stimulation efficacy was judged as swarming distance of emergent flares from colony edges compared with a positive control (as described in the Fig. 2D and E legends). Wild-type stimulation, >75% efficacy; weak stimulation, ∼20% efficacy; very weak stimulation, <5% efficacy. See Table S1 for strain details.

Sequence comparisons showed that S. cellulosum TraA orthologs lacked the C2 and C3 Cys-rich repeats (∼20 amino acids each) that are present in the TraA proteins from *Myxococcus* isolates (Fig. 1B; see also Fig. 3A and Fig. S4). Their absence raised the issue of whether these repeats were required for TraA function. We hypothesized that the Cys-rich repeats form a “stalk” to help display the recognition domain (VD) on the cell surface (4). To test for functional requirement of these and other Cys-rich repeats, we constructed a series of 11 in-frame deletions in TraA (Fig. 3B). As suggested from the S. cellulosum sequences, the first four tandem Cys-rich repeats were all individually dispensable, and, in fact, when two and even three of these repeats (but not four repeats) were deleted, full function was retained in the stimulation assay. In contrast, the distal repeats were all essential or nearly so for TraA function (Fig. 3B). We also identified an invariant sequence (SCNCCP) within tandem repeat C5 that is present in all 90 TraA orthologs (Fig. S5), suggesting that it plays a critical role. Supporting this notion, deletion of C5 abolished TraA function (Fig. 3B).

10.1128/mBio.02751-18.4 FIG S4 Sequence comparison of TraA^Soce_MSr7282^ and TraA^Mxx_DK1622^. Domains are shaded as indicated. Download FIG S4, TIF file, 1.1 MB. Copyright © 2019 Cao et al. 2019 Cao et al. This content is distributed under the terms of the Creative Commons Attribution 4.0 International license.

10.1128/mBio.02751-18.5 FIG S5 Invariant adjacent cysteine residues among all TraA orthologs. The domain architecture is shown at the top. The alignment of a subregion (encompassing C4 and C5) of diverse TraA orthologs is shown at the bottom. Only one representative ortholog from each myxobacterial species is included for simplicity. Invariant sequence SCNCCP is shaded. Download FIG S5, TIF file, 0.7 MB. Copyright © 2019 Cao et al. 2019 Cao et al. This content is distributed under the terms of the Creative Commons Attribution 4.0 International license.

### TraA recognition diversity among *Myxococcales* isolates.

**FIG 4:**
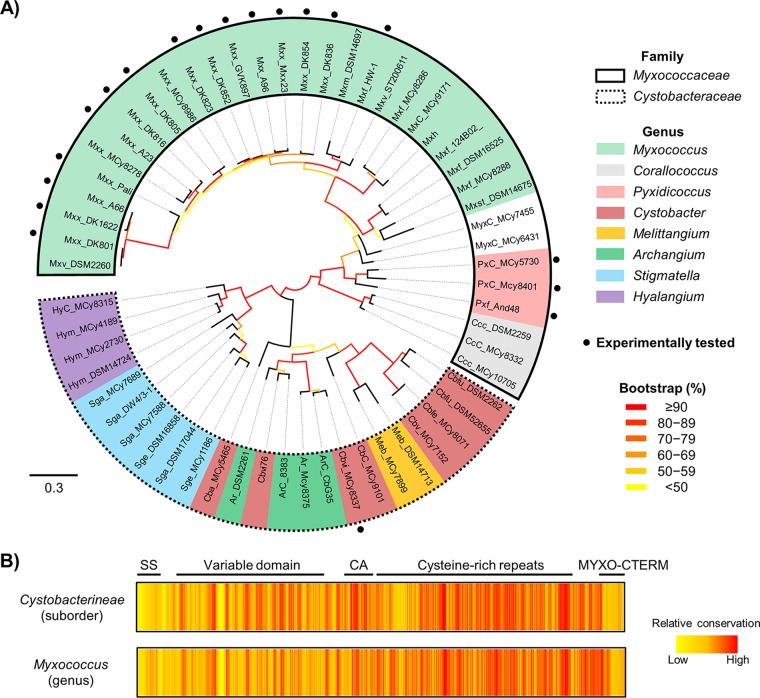
Bioinformatic analyses of TraA orthologs from the suborder *Cystobacterineae*. (A) Maximum likelihood tree showing the relationships among diverse *Cystobacterineae* TraA orthologs (full length). Families and genera are indicated by outlines and colors, respectively. Each allele prefix indicates its taxonomic origin (see Table 1 for details). TraA orthologs that were functionally characterized are marked with black dots. The scale bar represents the number of amino acid substitutions per residue. Bootstrap values (%) are color coded. (B) Heat maps showing sequence conservation of TraA orthologs from the suborder *Cystobacterineae* and the genus *Myxococcus*. TraA domain organization is indicated.

**FIG 5:**
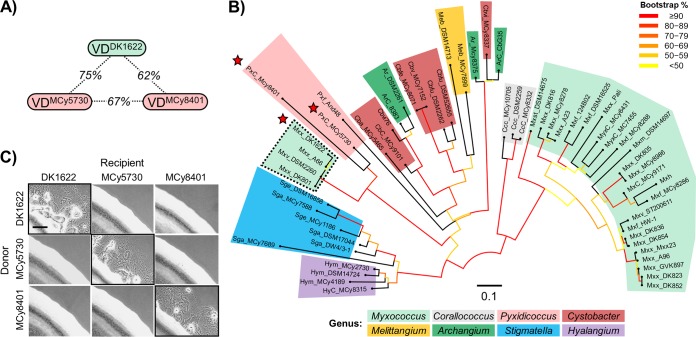
TraA orthologs harboring related VDs display allele-specific recognition. (A) Pairwise amino acid sequence identity among three related VDs. (B) Maximum likelihood tree of the VDs of 59 *Cystobacterineae* TraA orthologs. Red stars highlight the three related VDs shown in panel A. Each allele prefix shows the taxonomic origin (see Table 1 for details). Different genera are indicated by colors. The dashed border indicates members of recognition group A. Bootstrap values are indicated with color. The scale bar represents the number of substitutions per amino acid site. (C) Stimulation assays showing specific recognition among these *traA* alleles. Strain details are given in Table 2 (see also Table S1). Scale bar, 200 µm.

The VD in TraA dictates self-recognition among M. xanthus isolates (5). We tested whether this principle held true for TraA orthologs identified across the *Myxococcales* order. Because our prior work focused on TraA from the *Cystobacterineae* suborder, we started our analysis with this group. A phylogenetic tree of 59 full-length TraA orthologs from *Cystobacterineae* showed that TraA forms distinct clades that correlate with their taxonomic groupings (Fig. 4A). In addition, sequence analyses revealed that these TraA orthologs are highly divergent within their N-terminal regions, whereas their C-terminal regions are relatively conserved (Fig. 4B), supporting our findings that the VD confers recognition selectivity. Next, we experimentally analyzed two orthologs, TraA^MCy8401^ and TraA^MCy5730^, that contain VDs that are the most similar to the TraA from our M. xanthus DK1622 laboratory strain (5) (Fig. 5A and B). We note that the phylogenetic groupings of the VDs differ from the groupings of full-length TraA; e.g., we found that TraA^DK1622^ and the corresponding recognition group A members no longer resided within the *Myxococcus* clade (Fig. 5B). This suggests that horizontal gene transfer had contributed to the diversification of the VD. The interactions among these three alleles were analyzed experimentally by a stimulation assay, and they displayed three distinct recognition specificities (Fig. 5C), indicating that TraA governs self-recognition beyond the *Myxococcus* clade (Fig. 4A).

**FIG 6:**
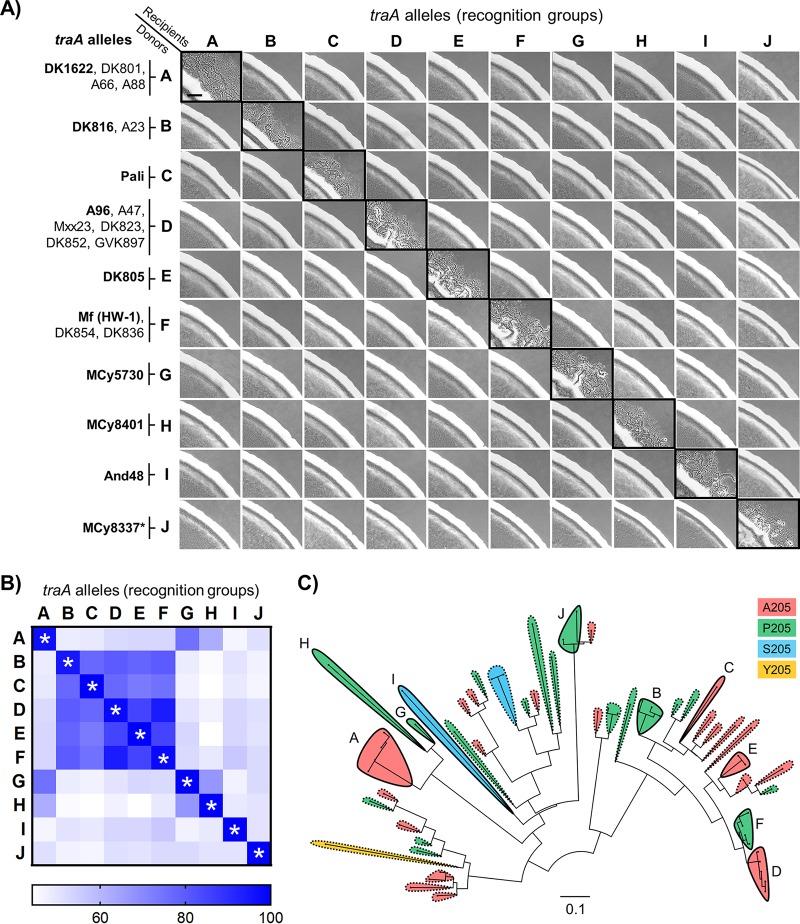
Self-recognition among a wide range of myxobacteria is governed by the *traA* locus. (A) Stimulation assays showing specific recognition among 10 *traA* alleles (names are shown in bold on the left) in an isogenic set of strains. Black borders highlight 10 distinct recognition groups (groups A to J). Additional group members that have been functionally characterized (5, 10) are also listed on the left. The asterisk indicates a chimeric allele harboring VD^MCy8337^ (see Fig. S3 for details). Scale bar, 200 µm. (B) Pairwise plot of (%) identity among VDs of the TraA orthologs tested in panel A (asterisks indicate self-recognition). (C) Same tree as that shown in Fig. 5B, where allele names are given. Shaded areas highlight distinct recognition groups, solid lines indicate characterized recognition groups (letters indicate group names), and dashed lines show predicted recognition groups. Groups are color coded according to the specificity-determining residue at position 205. The scale bar indicates the number of substitutions per amino acid residue.

To explore the phylogenetic breadth of TraA recognition across the order *Myxococcales*, we expanded this analysis to the distant orthologs TraA^And48^ and TraA^MCy8337^. Their VDs display 46% and 51% identity to the VD of TraA^DK1622^, respectively. Using the stimulation assay, we systematically tested the recognition specificity among the aforementioned four new environmental alleles and representative *traA* alleles from our prior groupings (5, 10). Pairwise interactions between isogenic donor and recipient strains bearing a collection of *traA* alleles revealed that they fall into 10 distinct recognition groups (Fig. 6A; strain details are given in Table 2). This result shows that *traA* alleles from a wide range of myxobacterial isolates display diverse specificities in cell-cell recognition.

### The A/P205 molecular switch.

The malleable nature of TraA may have facilitated its diversification into distinct recognition groups (10). For instance, we previously identified a molecular switch at residue 205 [according to TraA^Mf(HW-1)^ numbering] within the VD (10). Single substitutions (e.g., A205→P or P205→A) can reprogram the selectivity of cell-cell recognition and OME. Notably, residue A/P205 is highly conserved across all TraA alleles (Fig. S1), and closely related alleles typically contain the same residue, suggesting that this molecular switch may broadly influence recognition among diverse isolates. Within this larger collection of alleles, there were, however, rare exceptions in which a serine (S) or tyrosine (Y) was located at the position corresponding to A/P205. To assess the potential roles of these alternative residues in recognition, we used TraA receptors that harbor an A or P at position 205 as templates to replace the original residue with S or Y. On the basis of results from a stimulation assay, S205 showed specificity similar to that shown by A205 (Fig. S6A), suggesting that S205 and A205 display similar TraA conformations involved in recognition. In contrast, Y substitutions at A205 or P205 abolished TraA function (Fig. S6B), indicating that Y205 might be tolerated in only a limited number of alleles. Despite the key role of A/P205, other residues are also involved in TraA specificity. Further studies are needed to elucidate the molecular and structural basis in which various VD residues govern TraA recognition.

10.1128/mBio.02751-18.6 FIG S6 The roles of S205 and Y205 in TraA recognition. TraA orthologs from Mf and A96 were used as templates for the construction of single amino acid substitutions. (A and B) The specificity determinant residues of TraA^Mf^ (P205) and TraA^A96^ (A205) were changed to S205 (A) and Y205 (B). The effects of such substitutions on TraA function/recognition were analyzed by stimulation assays. The black borders in panel A highlight that S205 exhibited the same specificity as A205. A cartoon illustration of how single residue substitutions changed the recognition specificity of TraA is shown at the bottom. The black borders in panel B show that Y205 substitutions abolished TraA function. Strain details are given in Table S1. Scale bar, 200 µm. Download FIG S6, TIF file, 1.3 MB. Copyright © 2019 Cao et al. 2019 Cao et al. This content is distributed under the terms of the Creative Commons Attribution 4.0 International license.

Finally, we sought to estimate how many distinct recognition groups were present in our collection of *Cystobacterineae* alleles. We first calculated the pairwise identities between VDs of all the *traA* alleles that were functionally characterized (Fig. 6B). Among the *traA* alleles that belonged to the same recognition groups, the VD identities ranged from 95% to 100%. In addition, compatible alleles contained no indels within their VDs and harbored the same residue at position 205. In contrast, VD identities in different (experimentally defined) recognition groups typically range from 45% to 85%, can contain different residues at position 205, and can contain indels. It is also worth noting that the recognition incompatibility between groups D and F is due to a single residue difference at position 205 (10) (Fig. S6), even though the sequence identity of their VDs can be as high as 94%. On the basis of these findings, we considered two uncharacterized *traA* alleles to be compatible if their VDs (i) displayed ≥90% identity (a conservative value between the experimentally determined values of 95% and 85%), (ii) contained no indels, and (iii) harbored identical residues at position 205. From these assumptions, we inferred that the 59 TraA orthologs from *Cystobacterineae* fall into 42 distinct recognition groups (Fig. 6C). In addition, we conducted similar analyses on S. cellulosum TraA alleles. As shown in Fig. S7, TraA S. cellulosum sequences were also polymorphic within their VDs, implying that they are involved in molecular recognition. Using the criteria outlined above, we predict that the 22 TraA S. cellulosum orthologs form 12 distinct recognition groups. These combined analyses highlight the polymorphisms and diversity of TraA receptors in homotypic self-recognition among wild populations of myxobacteria.

10.1128/mBio.02751-18.7 FIG S7 Prediction of recognition diversity among S. cellulosum TraA orthologs. (A) Heat map showing sequence conservation of 22 TraA alleles from S. cellulosum. (B) Maximum likelihood tree of the VDs from 22 S. cellulosum TraA orthologs. Dashed border lines highlight predicted recognition groups. All of the S. cellulosum TraA alleles harbored a P at position 205. Branch support data are color coded. The scale bar indicates the number of substitutions per amino acid residue. Download FIG S7, TIF file, 0.7 MB. Copyright © 2019 Cao et al. 2019 Cao et al. This content is distributed under the terms of the Creative Commons Attribution 4.0 International license.

## DISCUSSION

Here we provide bioinformatic and experimental evidence that a single *traA* locus is sufficient to determine the recognition specificity for the sharing of cellular goods across a broad phylogenetic range of myxobacteria. Examples of single alleles governing social interactions have been described previously in a few other microbial species. For example, *FLO1* promotes flocculation in yeasts, and, under certain conditions, only cells bearing this adhesin can congregate into flocs (29). However, the sequence variability of *FLO1* among yeast isolates is limited and most likely determines only the competency of flocculation rather than partner specificity (29, 30). In Dictyostelium discoideum (31) and Proteus mirabilis (32), specific recognition is achieved through heterotypic interactions between two variable proteins. In contrast, our report provides a rare example in which a single polymorphic receptor is sufficient to modulate specificity in social behaviors through homotypic interactions.

We previously showed that variable TraA receptors enable cells to adhere differentially to only those cells that bear the identical receptor (10), demonstrating that homotypic binding governs selectivity. We also hypothesize that single-residue (A/P205) changes in TraA alter the conformation of a loop and hence recognition specificity (10). How TraA sequence variation generates many different binding interfaces that allow specificity in recognition remains an intriguing question. As mentioned, although TraB does not contribute toward specificity, it is required to form a functional adhesin with TraA and for OME.

What are the sources of genetic diversity within the *traA* locus? We suspect that the malleable nature of TraA has enabled it to tolerate *de novo* mutations that explore different conformations involved in homotypic recognition specificity, which in turn has led to distinct recognition groups. In addition to spontaneous mutations, horizontal gene transfer of *traA* has been suggested to occur between isolates (5). Horizontal acquisition of new alleles and homologous recombination between alleles likely contribute to genetic variability in *traA*. One example in which allele shuffling may have occurred is shown in Fig. 5B. Here, members from recognition group A formed a clade based on their VD sequences with the *Pyxidicoccus* isolates. However, when the full TraA sequence was used for analysis, group A members instead formed a clade with other *Myxococcus* isolates (Fig. 4A). In contrast to the high level of polymorphisms found within the VD, the C-terminal region of TraA displays less sequence variability. This region likely has conserved and essential roles in OME that restrict sequence variation.

How polymorphisms at a greenbeard locus are selected and maintained during evolution is an interesting puzzle (2, 14, 15). Wild populations of myxobacteria display extensive strain diversity, including at the subspecies level (33, 34). TraA polymorphisms are likely to have a role in creating social barriers that limit the cooperative behavior exemplified by sharing of cellular goods with clonemates and avoid possible adverse interactions with nonself cells (7, 8). As a greenbeard gene, *traA* itself determines OME compatibilities between cells, regardless of genetic relatedness at other loci. However, myxobacteria also contain a second discrimination layer to ensure that cells engaged in OME are truly clonemates or at least have a recently shared common ancestor (7). That is, a suite of polymorphic lipoprotein toxins are exchanged between cells bearing compatible TraA receptors, along with the bulk exchange of lipids and other proteins. Distinct isolates harbor different repertoires of *tra*-dependent polymorphic toxins and antitoxins, the latter of which are not transferred. Consequently, partners involved in OME must produce cognate antitoxins to transferred toxins to avoid a lethal outcome, which indeed occurs during OME between clonemates. We hypothesize that this antagonism provides selective pressure that drives and maintains *traA* allele diversity in wild populations. For example, the antagonistic interactions between cells bearing compatible TraA receptors would favor the selection of variants that display different specificities and would thus avoid antagonism with the dominant population. In turn, a novel *traA* recognition allele allows those individuals to cooperate among themselves and insulates them from detrimental interactions with other populations.

The wide distribution of *traAB* genes within the *Myxococcales* order supports the idea of the prevalence of OME among diverse isolates. OME is likely beneficial to myxobacteria as a consequence of promoting cooperation among clonemates and antagonism toward nonkin, which in turn promotes population diversity and resilience (6, 7, 35). Given the important roles of OME in modulating social behaviors, we were struck by the fact that some myxobacteria, e.g., C. crocatus, lack *traAB*. One obvious reason why some species lack *traAB* is that the exchange of toxins creates intense interstrain competition. This antagonism is alleviated by removing TraA/B function, and, indeed, this rapidly occurs when two strains compete by OME under laboratory conditions (8, 36). In addition, OME is energetically costly because it involves OM fusion and bulk cellular exchange. Loss of OME may thus provide fitness benefits under certain conditions. Finally, as noted above, *Anaeromyxobacter* and *Vulgatibacter* isolates also lack *traAB*, which may be related to their smaller genomes and is consistent with the absence of a number of social traits in these species (20–23).

The TraA/B proteins from S. cellulosum were not functional under conditions of heterologous expression in M. xanthus. Given the unique OM lipid composition of S. cellulosum (28), we suspect that their TraA/B proteins evolved to function in a different physiochemical environment that is not compatible with OME function in the M. xanthus OM. However, the TraA VDs of S. cellulosum isolates are also highly polymorphic (see Fig. S7 in the supplemental material), suggesting that they function in molecular recognition. Given that the S. cellulosum TraA/B domain architecture is like that of other TraA/B proteins (Fig. 3A), the S. cellulosum TraA/B proteins likely reside on the cell surface and function as adhesins. Nevertheless, further studies are needed to elucidate the function of TraA/B in S. cellulosum and to test whether OME is a regulated process that occurs only under certain conditions or during specific developmental stages.

Taken together, our results have shown the prevalence and diversity of the *traAB* genes, and hence the widespread occurrence of OME, in the order *Myxococcales*. From the analyses of the 90 TraA sequences, we predict that they represent >60 distinct TraA recognition groups (42 from *Cystobacterineae*, 12 from S. cellulosum, and the remaining number from other taxonomic groups shown in Fig. S1). To our knowledge, there are no other loci involved in cooperative social interactions that offer the diversity in recognition that has been reported here for TraA. Given that the extent of *Myxococcales* taxonomic diversity is currently unknown but clearly is vast (17, 33), our 90 representative alleles thus represent a great underestimation of the global diversity of *traA*. On the basis of this, we think there are many more distinct TraA recognition groups in nature that influence social interactions within the *Myxococcales* order.

## MATERIALS AND METHODS

### Bacterial strains and growth conditions.

Bacterial strains used in this study are listed in Table S1 in the supplemental material. M. xanthus was cultured in CTT medium (1% [wt/vol] Casitone, 10 mM Tris-HCl [pH 7.6], 1 mM KH_2_PO_4_, 8 mM MgSO_4_) in the dark at 33°C with shaking. S. cellulosum was maintained on VY/2 (37) agar medium in the dark at 33°C. To propagate S. cellulosum in liquid culture, soluble medium M [0.5% soy peptone, 1% maltose-monohydrate, 0.1% CaCl_2_·2H_2_O, 0.1% MgSO_4_·7H_2_O, 22 µM EDTA iron(III) sodium salt, 1.2% HEPES, pH 7.2] (38) was used. Escherichia coli was cultured in LB at 37°C. To generate solid medium for plate culture, 1.5% (wt/vol) agar (Criterion agar; Hardy Diagnostics) was added to the medium. For selection, 50 µg/ml kanamycin (Km) and 50 µg/ml zeocin (Zeo) were added to the media as needed.

### Searches for *traAB* orthologs.

BLASTP searches were performed to identify orthologs of *traAB* across the *Myxococcales* order. Sequences of the TraA and TraB proteins from M. xanthus DK1622 were used as queries. The *traAB* orthologs with E values of <1 × 10^−100^ and query coverage values of >80% were retained for further analysis. In addition to searching for orthologs in the nonredundant NCBI protein database and the integrated microbial genomes (IMG) database (39), we also searched myxobacterial genomes from our in-house database (Müller laboratory) by following the same criteria. Next, we determined whether these sequences were *traAB* orthologs by (i) comparing their predicted domain architectures to that of TraA/B^DK1622^ and (ii) confirming that the corresponding parental strains harbored both *traA* and *traB* orthologs. In all cases examined, the corresponding genes overlapped in an operon. We also included an additional 12 TraA sequences from our prior work (5), in which *traB* was not sequenced. In one case (Cbfu_MCy9118), only a TraB ortholog was identified, probably because the genome sequence was not complete. Finally, we manually inspected each ortholog to correct for annotation errors, particularly analyzing whether the most likely start codon had been chosen. The final sequence files included 90 TraA orthologs and 79 TraB orthologs.

### Bioinformatic analysis.

Sequences of TraA/B orthologs were aligned in MUSCLE (40). To create Fig. 1B, the alignments were visualized in Jalview (41) using the Clustal X default color scheme. To determine the boundaries of the VDs, orthologs were compared to TraA^DK1622^ in alignments. The VD in TraA^DK1622^ encompasses amino acids 62 to 259, and the sequences of other TraA orthologs were trimmed accordingly.

For phylogenetic analyses, maximum likelihood (ML) phylogenetic trees were constructed in PhyML 3.0 (42). We first predicted the best-fitting evolutionary models based on the corrected Akaike information criterion in ProtTest 3 (43). To generate phylogeny for the 59 TraA orthologs from the *Cystobacterineae* suborder (Fig. 4A), the best model (WAG+I+G+F) chosen by ProtTest was used, and 1,000 bootstrap replicates were performed. Regions encompassing the VDs of *Cystobacterineae* TraA orthologs were also used to generate a ML tree (Fig. 5B; see also Fig. 6C), based on a JTT+I+G model and 1,000 bootstrap replicates. We constructed a phylogenetic tree for the VDs of S. cellulosum TraA orthologs by using a WAG+I+G model and 1,000 bootstrap replicates (see Fig. S7 in the supplemental material). In addition, all the TraA and TraB sequences from the *Myxococcales* order were used to generate ML trees (Fig. S1) using a WAG+I+G model (300 bootstrap replicates) and a LG+I+G+F model (500 bootstrap replicates), respectively. Phylogenetic trees were visualized in FigTree v1.4.3 (http://tree.bio.ed.ac.uk/software/figtree/).

To analyze residue conservation of diverse TraA orthologs, we first aligned the desired sequences (alignment gaps were removed) and scored residue conservation using Jensen-Shannon divergence (44). The corresponding heat maps were generated in Microsoft Excel. Protein sequence similarity analyses were conducted using SIM (45). A heat map showing the pairwise sequence identity of diverse VDs (Fig. 6B) was generated in Prism GraphPad (GraphPad Software, Inc.).

### Plasmid and strain construction.

Plasmids and primers used in this study are listed in Table S1 and Table S2. The *traA-* and *traB-*containing plasmids (pPC26 to pPC35) were constructed by inserting appropriate alleles downstream of the *pilA* promoter (P*_pilA_*) in the pDP22 vector (between XbaI and HindIII) as described previously (10). To generate the *traA* chimeric allele (pPC36), desired subregions of *traA*^DK1622^ and *traA*^MCy8337^ were ligated into pDP22 (linearized by EcoRI and XbaI digestion) using Gibson Assembly Master Mix (New England Biolabs) as described previously (10). Site-directed mutagenesis was done by Gibson assembly as described previously (10) to generate pPC37-40. To create markerless in-frame deletions within the Cys-rich region of TraA, different *traA* fragments harboring the desired deletions were synthesized (GenScript) and subcloned into *traA-*containing plasmid pDP27 (linearized using SacI and NgoMIV or NgoMIV and HindIII) to create pXW8 to pXW11 and pXW15 to pXW17. The rest of the Cys-rich deletion mutants (pXW12 to pXW14 and pXW18) were generated through Gibson assembly. To create pXW7, the *neoR*/*kanR* genes were first removed from pCR-XL-TOPO (Invitrogen) by PCR and blunt-end self-ligation. Subsequently, *traB*^DK1622^ and a fragment for integration at the Mx9 attachment site were introduced into the multiple cloning sites of this plasmid.

10.1128/mBio.02751-18.9 TABLE S2 Primers used in this study. Download Table S2, DOCX file, 0.03 MB. Copyright © 2019 Cao et al. 2019 Cao et al. This content is distributed under the terms of the Creative Commons Attribution 4.0 International license.

All plasmids were verified by PCR, restriction enzyme digestion and/or by DNA sequencing. Plasmids were then transformed into M. xanthus cells and selected with appropriate antibiotics. All cloning vectors were integrated at the Mx8 attachment site of the M. xanthus genome, except for pXW7 (Mx9 attachment site).

### Stimulation assay.

This assay was done as described previously (10). Briefly, M. xanthus cells were cultured overnight to mid-log phase, washed, and resuspended in TPM buffer (10 mM Tris-HCl [pH 7.6], 1 mM KH_2_PO_4_, 8 mM MgSO_4_) to a calculated density of ∼2.5 × 10^9^ cells/ml. Mixtures (1:1 ratio) of desired strain combinations were then spotted onto 1/2 CTT (Casitone reduced to 0.5%) agar plates supplemented with 2 mM CaCl_2_. The edges of colonies were imaged after overnight incubation at 33°C.

Stimulation efficacy was estimated by measuring the distance of each swarm of emergent flares from the inoculum edges. Cell mixtures subjected to this analysis were spotted onto the same agar plate and incubated for the same time before imaging. The averaged swarm distance (*d*_allele_) was determined for each colony in ImageJ software (https://imagej.nih.gov/ij/). Swarm distances (*d*_allele_) of different mixtures were normalized against the swarm distance of the control group (*d*_DK1622_) on the same agar plate. Four experimental replicates were performed. Data analyses, including unpaired and two-tailed *t* tests, were done in Prism GraphPad.

### Transfer assay.

We developed a method to label myxobacterial OM proteins with Cy3 (Lumiprobe) to test for transfer between cells. Live cells were grown to mid-log phase, washed, and incubated with Cy3 dyes in appropriate liquid medium as follows: for M. xanthus, 50 µg/ml Cy3 and 1-h incubation in CTT; for S. cellulosum, 1 µg/ml Cy3 and 15-min incubation in medium M. After incubation, the stained cells were washed three to five times in fresh medium and resuspended to ∼5 × 10^8^ cells/ml. Recipient cells were stained with carboxyfluorescein diacetate succinimidyl ester (CFDA-SE; Invitrogen) as described previously (5). Cy3-labeled donors and CFDA-SE-labeled recipients were then mixed (1:1 ratio) and incubated on agarose pads consisting of 1.2% agarose in the appropriate medium. Cell mixtures were incubated in the dark at 33°C for 0.5 h for M. xanthus cells and for ≥4 h for S. cellulosum cells before imaging was performed. To test for OME in C. crocatus Cm c5, a lipid dye transfer assay was done as described previously (9). Donor cells were labeled with a red fluorescent DiD lipid dye (lipophilic tracer sampler kit; Invitrogen), whereas the recipients were labeled with CFDA-SE (Invitrogen).

### Microscopy.

Images of stimulation experiments were acquired using a Nikon E800 phase contrast/fluorescence microscope equipped with a 10× lens objective. Fluorescence microscopy of transfer assays was done with a 60× oil lens objective and fluorescein isothiocyanate (FITC) or Texas Red filter sets.

### Data accessibility.

The new *traAB* DNA sequences used in this study have been deposited into the GenBank database under accession numbers MK091159 to MK091255.
