# Supplementary material for: A Highly Polymorphic Receptor Governs Many Distinct Self-Recognition Types within the Myxococcales Order
Source: mBio. 2019 Feb 12;10(1):e02751-18. doi: 10.1128/mBio.02751-18 (PMC6372800; doi:10.1128/mBio.02751-18)
Supplement: TABLE S1 [file mBio.02751-18-st001.docx]

**Table S1. Plasmids and strains used in this study.**

| **Plasmids** | **Relevant features** | | **Source** |
| --- | --- | --- | --- |
| pDP22 | P*_pilA_* in pSWU19 (Mx8 *attP*), Km^r^ | | (1) |
| pDP23 | P*_pilA_*-*traA*^DK816^ in pSWU19, Km^r^ | | (1) |
| pDP24 | P*_pilA_*-*traA*^A96^ in pSWU19, Km^r^ | | (1) |
| pDP25 | P*_pilA_*-*traA^Mf^* in pSWU19, Km^r^ | | (1) |
| pDP26 | P*_pilA_*-*traA*^Pali^ in pSWU19, Km^r^ | | (1) |
| pDP27 | P*_pilA_*-*traA*^DK1622^ in pSWU19, Km^r^ | | (1) |
| pPC16 | P*_pilA_*-*traA*^DK805^ in pSWU19, Km^r^ | | (2) |
| pPC26 | P*_pilA_*-*traA*^MCy5730^ in pSWU19, Km^r^ | | This study |
| pPC27 | P*_pilA_*-*traA*^MCy8401^ in pSWU19, Km^r^ | | This study |
| pPC28 | P*_pilA_*-*traA*^And48^ in pSWU19, Km^r^ | | This study |
| pPC29 | P*_pilA_*-*traA*^MCy8337^ in pSWU19, Km^r^ | | This study |
| pPC30 | P*_pilA_*-*traA*^MCy7282^ in pSWU19, Km^r^ | | This study |
| pPC31 | P*_pilA_*-*traB*^MCy5730^ in pSWU19, Km^r^ | | This study |
| pPC32 | P*_pilA_*-*traB*^MCy8401^ in pSWU19, Km^r^ | | This study |
| pPC33 | P*_pilA_*-*traB*^And48^ in pSWU19, Km^r^ | | This study |
| pPC34 | P*_pilA_*-*traB*^MCy8337^ in pSWU19, Km^r^ | | This study |
| pPC35 | P*_pilA_*-*traB*^MSr7282^ in pSWU19, Km^r^ | | This study |
| pPC36 | P*_pilA_*-*traA*^MCy8337/DK1622^ in pSWU19, Km^r^ | | This study |
| pPC37 | P*_pilA_*-*traA*^Mf^ (P205S) in pSWU19, Km^r^ | | This study |
| pPC38 | P*_pilA_*-*traA*^A96^ (P205S) in pSWU19, Km^r^ | | This study |
| pPC39 | P*_pilA_*-*traA*^Mf^ (P205Y) in pSWU19, Km^r^ | | This study |
| pPC40 | P*_pilA_*-*traA*^A96^ (P205Y) in pSWU19, Km^r^ | | This study |
| pCR®-XL-TOPO® | Cloning vector, Km^r^, Zeo^r^ | | Invitrogen |
| pXW7 | P*_pilA_*-*traB* in pCR®-XL-TOPO® (Δ*neoR/kanR*, Mx9 *attP*)*,* Zeo^r^ | | This study |
| pXW8 | P*_pilA_*-*traA*^DK1622^ (ΔCA) in pSWU19, Km^r^ | | This study |
| pXW9 | P*_pilA_*-*traA*^DK1622^ (ΔC1) in pSWU19, Km^r^ | | This study |
| pXW10 | P*_pilA_*-*traA*^DK1622^ (ΔC2-3) in pSWU19, Km^r^ | | This study |
| pXW11 | P*_pilA_*-*traA*^DK1622^ (ΔC4) in pSWU19, Km^r^ | | This study |
| pXW12 | P*_pilA_*-*traA*^DK1622^ (ΔC2-4) in pSWU19, Km^r^ | | This study |
| pXW13 | P*_pilA_*-*traA*^DK1622^ (ΔC1-4) in pSWU19, Km^r^ | | This study |
| pXW14 | P*_pilA_*-*traA*^DK1622^ (ΔC5) in pSWU19, Km^r^ | | This study |
| pXW15 | P*_pilA_*-*traA*^DK1622^ (ΔC6E) in pSWU19, Km^r^ | | This study |
| pXW16 | P*_pilA_*-*traA*^DK1622^ (ΔC7E) in pSWU19, Km^r^ | | This study |
| pXW17 | P*_pilA_*-*traA*^DK1622^ (ΔC8E) in pSWU19, Km^r^ | | This study |
| pXW18 | P*_pilA_*-*traA*^DK1622^ (ΔC9E) in pSWU19, Km^r^ | | This study |
| **Strains** | **Relevant features*** | **Experimental use** | **Source** |
| DH5α | *E. coli* cloning strain |  | Lab collection |
| DK1622 | Wild-type *M. xanthus,* motile |  | (3) |
| Soce_MSr7237 | a.k.a. Soce377, *S. cellulosum* isolate | Fig. S2A | Müller lab |
| So0157-24 | *S. cellulosum* isolate | Fig. S2A | Li lab |
| Cm c5 | *Chondromyces* *crocatus* isolate | Fig. S2B | Müller lab |
| DK8601 | *aglB1* (*aglQ1*) Δ*pilA*::tc, nonmotile, Tc^r^ | Stimulation donor | (4) |
| DK8615 | DK1622 Δ*pilQ* (markerless) | Fig. S2A | (5) |
| DW1415 | DK8615 *traA*::km, Km^r^ | Fig. S2A | (6) |
| DW1466 | DK1622 Δ*tgl*::tc Δ*cglC* (markerless), nonmotile, Tc^r^ | Stimulation recipient | (6) |
| DW1467 | DK8601 Δ*traA* (markerless), Tc^r^ | Δ*traA* stimulation donor | (1) |
| DW1485 | DK8601 Δ*traB* (markerless), Tc^r^ | Δ*traB* stimulation donor | This study |
| DW1483 | DK8601 Δ*traAB* (markerless), Tc^r^ | Δ*traAB* stimulation donor | (2) |
| DW2220 | DW1466 Δ*traA* (markerless), Tc^r^ | Δ*traA* stimulation recipient | (2) |
| DW2243 | DW1467 (pPC26), Km^r^, Tc^r^ | Fig. 2C, 5C, and 6A | This study |
| DW2244 | DW1467 (pPC27), Km^r^, Tc^r^ | Fig. 2C, 5C, and 6A | This study |
| DW2245 | DW1467 (pPC28), Km^r^, Tc^r^ | Fig. 2C, and 6A | This study |
| DW2246 | DW1467 (pPC29), Km^r^, Tc^r^ | Fig. 2C | This study |
| DW2247 | DW1467 (pPC30), Km^r^, Tc^r^ | Fig. 2C | This study |
| DW2248 | DW2220 (pPC26), Km^r^, Tc^r^ | Fig. 2C, 5C, and 6A | This study |
| DW2249 | DW2220 (pPC27), Km^r^, Tc^r^ | Fig. 2C, 5C, and 6A | This study |
| DW2254 | DW2220 (pPC28), Km^r^, Tc^r^ | Fig. 2C, and 6A | This study |
| DW2255 | DW2220 (pPC29), Km^r^, Tc^r^ | Fig. 2C | This study |
| DW2256 | DW2220 (pPC30), Km^r^, Tc^r^ | Fig. 2C | This study |
| DW2257 | DW1485 (pPC31), Km^r^, Tc^r^ | Fig. 2D | This study |
| DW2258 | DW1485 (pPC32), Km^r^, Tc^r^ | Fig. 2D | This study |
| DW2259 | DW1485 (pPC33), Km^r^, Tc^r^ | Fig. 2D | This study |
| DW2260 | DW1485 (pPC34), Km^r^, Tc^r^ | Fig. 2D | This study |
| DW2261 | DW1485 (pPC35), Km^r^, Tc^r^ | Fig. 2D | This study |
| DW2262 | DW1467 (pPC36, pXW7), Km^r^, Tc^r^, Zeo^r^ | Fig. 6A, and S3 | This study |
| DW2263 | DW2220 (pPC36, pXW7), Km^r^, Tc^r^, Zeo^r^ | Fig. 6A and S3 | This study |
| DW1194 | DW1467 (pXW8), Km^r^, Tc^r^ | Fig. 3 | This study |
| DW1195 | DW1467 (pXW9), Km^r^, Tc^r^ | Fig. 3 | This study |
| DW1196 | DW1467 (pXW10), Km^r^, Tc^r^ | Fig. 3 | This study |
| DW1197 | DW1467 (pXW11), Km^r^, Tc^r^ | Fig. 3 | This study |
| DW1198 | DW1467 (pXW12), Km^r^, Tc^r^ | Fig. 3 | This study |
| DW1199 | DW1467 (pXW13), Km^r^, Tc^r^ | Fig. 3 | This study |
| DW1200 | DW1467 (pXW14), Km^r^, Tc^r^ | Fig. 3 | This study |
| DW2000 | DW1467 (pXW15), Km^r^, Tc^r^ | Fig. 3 | This study |
| DW2001 | DW1467 (pXW16), Km^r^, Tc^r^ | Fig. 3 | This study |
| DW2002 | DW1467 (pXW17), Km^r^, Tc^r^ | Fig. 3 | This study |
| DW2003 | DW1467 (pXW18), Km^r^, Tc^r^ | Fig. 3 | This study |
| DW1468 | DW1467 (pDP23), Km^r^, Tc^r^ | Fig. 6A | (1) |
| DW1469 | DW1467 (pDP24), Km^r^, Tc^r^ | Fig. 6A, and S6 | (1) |
| DW1470 | DW1467 (pDP25), Km^r^, Tc^r^ | Fig. 6A, and S6 | (1) |
| DW1471 | DW1467 (pDP26), Km^r^, Tc^r^ | Fig. 6A | (1) |
| DW2212 | DW1467 (pPC16), Km^r^, Tc^r^ | Fig. 6A | (2) |
| DW2221 | DW2220 (pDP23), Km^r^, Tc^r^ | Fig. 6A | (2) |
| DW2222 | DW2220 (pDP24), Km^r^, Tc^r^ | Fig. 6A, and S6 | (2) |
| DW2223 | DW2220 (pDP25), Km^r^, Tc^r^ | Fig. 6A, and S6 | (2) |
| DW2224 | DW2220 (pDP26), Km^r^, Tc^r^ | Fig. 6A | (2) |
| DW2234 | DW2220 (pPC16), Km^r^, Tc^r^ | Fig. 6A | (2) |
| DW2264 | DW2220 (pPC37), Km^r^, Tc^r^ | Fig. S6 | This study |
| DW2265 | DW2220 (pPC38), Km^r^, Tc^r^ | Fig. S6 | This study |
| DW2266 | DW1467 (pPC39), Km^r^, Tc^r^ | Fig. S6 | This study |
| DW2267 | DW1467 (pPC40), Km^r^, Tc^r^ | Fig. S6 | This study |
| DW2268 | DW2220 (pPC39), Km^r^, Tc^r^ | Fig. S6 | This study |
| DW2269 | DW2220 (pPC40), Km^r^, Tc^r^ | Fig. S6 | This study |

***** All “DW” and “DK” designated strains are derived from *M. xanthus* DK1622.

**References:**

1. Pathak, Darshankumar T., et al. "Molecular recognition by a polymorphic cell surface receptor governs cooperative behaviors in bacteria." *PLoS genetics* 9.11 (2013): e1003891.

2. Cao, Pengbo, and Daniel Wall. "Self-identity reprogrammed by a single residue switch in a cell surface receptor of a social bacterium." *Proceedings of the National Academy of Sciences* (2017): 201700315.

3. Dey, Arup, et al. "Sibling rivalry in Myxococcus xanthus is mediated by kin recognition and a polyploid prophage."*Journal of bacteriology* (2016): JB-00964.

4. Wall, Daniel, and Dale Kaiser. "Alignment enhances the cell-to-cell transfer of pilus phenotype." *Proceedings of the National Academy of Sciences* 95.6 (1998): 3054-3058.

5. Wall, Daniel, Paul E. Kolenbrander, and Dale Kaiser. "The Myxococcus xanthus pilQ (sglA) gene encodes a secretin homolog required for type IV pilus biogenesis, social motility, and development." *Journal of bacteriology* 181.1 (1999): 24-33.

6. Pathak, Darshankumar T., et al. "Cell contact–dependent outer membrane exchange in Myxobacteria: genetic determinants and mechanism." *PLoS genetics* 8.4 (2012): e1002626.
