# Supplementary material for: A Highly Polymorphic Receptor Governs Many Distinct Self-Recognition Types within the Myxococcales Order
Source: mBio. 2019 Feb 12;10(1):e02751-18. doi: 10.1128/mBio.02751-18 (PMC6372800; doi:10.1128/mBio.02751-18)
Supplement: TABLE S2 [file mBio.02751-18-st002.docx]

**Table S2 Primers used in this study.**

| **Primer name** | **Sequence (5’→3’)*** |
| --- | --- |
| TraA^MCy5730^-RBS-XbaI-F | GACGACTCTAGAGGAAACCAAGAATAGAAATAGAAAGGAGAATTAGTGCCCGATGATGTGCGCC |
| TraA^MCy5730^-HindIII-R | GACGACAAGCTTTCATCGAGCGCGGCGCTGGCTAC |
| TraA^MCy8401^-RBS-XbaI-F | GACGACTCTAGAGGAAACCAAGAATAGAAATAGAAAGGAGAATTAGTGCTCGATGATGTGCGCCC |
| TraA^MCy8401^-HindIII-R | GACGACAAGCTTTCATCGAGCGCGGCGCTGGCTAC |
| TraA^And48^-RBS-*Xba*I-F | GACGACTCTAGAGGAAACCAAGAATAGAAATAGAAAGGAGAATTAATGCTCGATGATGTGCGCCCCT |
| TraA^And48^-HindIII-R | GACGACAAGCTTTCATCGAGCGCGGCGCTGGCTAC |
| TraA^MCy8337^-RBS-XbaI-F | GACGACTCTAGAGGAAACCAAGAATAGAAATAGAAAGGAGAATTAATGGCCGCCCTGGCGGCGACGCTGCTGGTGA |
| TraA^MCy8337^-HindIII-R | GACGACAAGCTTTCACGCGGAGCCTCGCGGGCGGAG |
| TraA^MSr7282^-RBS-XbaI-F | GACGACTCTAGAGGAAACCAAGAATAGAAATAGAAAGGAGAATTAGTGCTGAAGAGCAGCGACCGAGC |
| TraA^MSr7282^-HindIII-R | GACGACAAGCTTTCACGACGGACCTCCGGCG |
| TraB^MCy5730^-RBS-XbaI-F | GACGACTCTAGAGGATCGACGCCTATATAGACGGGAGATATAATGAAGCCCTCGCACCTCTC |
| TraB^MCy5730^-HindIII-R | GACGACAAGCTTCTACGGCTTCGGCGCCCTGAG |
| TraB^MCy8401^-RBS-XbaI-F | GACGACTCTAGAGGATCGACGCCTATATAGACGGGAGATATAATGAAGCCCTCGCACCTCTC |
| TraB^MCy8401^-HindIII-R | GACGACAAGCTTCTACCGCTTCGGCGTGGAGG |
| TraB^And48^-RBS-XbaI-F | GACGACTCTAGAGGATCGACGCCTATATAGACGGGAGATATAATGAAGCCCTCGCACCTCTC |
| TraB^And48^-HindIII-R | GACGACAAGCTTCTACCGCTTCGGCGCCGTGGGCGCCTTGGGCTGCTTC |
| TraB^MCy8337^-RBS-XbaI-F | GACGACTCTAGAGGATCGACGCCTATATAGACGGGAGATATAGTGAAGCGCTTCGCCCCC |
| TraB^MCy8337^-HindIII-R | GACGACAAGCTTCTACCGCTTGCCCTTGGTCGG |
| TraB^MSr7282^-RBS-XbaI-F | GACGACTCTAGAGGATCGACGCCTATATAGACGGGAGATATAGTGAGAAAGCGCCCGCTCC |
| TraB^MSr7282^-HindIII-R | GACGACAAGCTTCTACGGCTTCGGACCTGCAGG |
| pSWU19-EcoRI-P*_pilA_*-F | AGGAAACAGCTATGACCATGATTACGAATTCCGTCATGTTGGACGAGGT |
| SS^DK1622^ -R | CTTACGGCTCTGcgcctgcgcaacactggc |
| VD^MCy8337^-F | gttgcgcaggcgCAGAGCCGTAAGCCGCAA |
| VD^MCy8337^-R | gcggttgcactgCTCGCACCGTGCCGGATC |
| C-terminus^DK1622^-F | GCACGGTGCGAGcagtgcaaccgccagttc |
| pSWU19-XbaI-HindIII-TraA-R | tgcatgcctgcaggtcgactctagaAAGCTTGAAGAGCTGCACGTTGAAG |
| TraA^A96^ (A205S)-R | GCCACGTGCTGAATCCGAGCACCGGTGG |
| TraA^A96^ (A205S)-F | CCGGTGCTCGGATTCAGCACGTGGCGAACCACCAAC |
| TraA^A96^ (A205Y)-R | GCCACGTGTAGAATCCGAGCACCGGTGG |
| TraA^A96^ (A205Y)-F | CCGGTGCTCGGATTCTACACGTGGCGAACCACCAAC |
| TraA^Mf^ (A205S)-R | GCCACGTGCTGAACCCGAGCATCGGTGG |
| TraA^Mf^ (A205S)-F | CCGATGCTCGGGTTCAGCACGTGGCGAACCACCAAC |
| TraA^Mf^ (A205Y)-R | GCCACGTGTAGAACCCGAGCATCGGTGG |
| TraA^Mf^ (A205Y)-F | CCGATGCTCGGGTTCTACACGTGGCGAACCACCAAC |
| TraA (ΔC2-4)-R | GGGCGAGCACACGTTGGTTTCGAGGTCGCAGCG |
| TraA (ΔC2-4)-F | CCTCGAAACCAACGTGTGCTCGCCCTGCGCCACC |
| TraA (ΔC1-4)-R | GGGCGAGCACACGGTGCGGCCGTTGAGGTTGGC |
| TraA (ΔC1-4)-F | AACGGCCGCACCGTGTGCTCGCCCTGCGCCACC |
| TraA (ΔC5)-R | GGCACTGGGTGCCGTTGTCGGTGCAGGACTCG |
| TraA (ΔC5)-F | TGCACCGACAACGGCACCCAGTGCCTGGCGC |
| TraA (ΔC9E)-R | GGTGCCGCAGCAGCGCGTGGTGGGGTTGCAC |
| TraA (ΔC9E)-F | CCCCACCACGCGCTGCTGCGGCACCACCGCC |

*Restriction sites are underlined.
